# Strain Characterization of *Streptococcus suis* Serotypes 28 and 31, Which Harbor the Resistance Genes *optrA* and *ant(6)-Ia*

**DOI:** 10.3390/pathogens10020213

**Published:** 2021-02-16

**Authors:** Shujie Wang, Defu Zhang, Chenggang Jiang, Haijuan He, Chenchen Cui, Weitong Duan, Shouping Hu, Jun Wang, Xuehui Cai

**Affiliations:** 1 National Key Laboratory of Veterinary Biotechnology, Harbin Veterinary Research Institute, Chinese Academy of Agricultural Sciences, Harbin 150001, China; jcg5168@163.com (C.J.); 13115601921@163.com (H.H.); cuichenchen@163.com (C.C.); wtduan1993@163.com (W.D.); hushouping@caas.cn (S.H.); 2 College of Food Science and Technology, Bohai University, Jinzhou 121013, China; zhangdf@bhu.edu.cn; 3 Institute of Animal Husbandry, Heilongjiang Academy of Agriculture Sciences, Harbin 150086, China; 4 Beijing General Station of Animal Husbandry, Beijing 100029, China; wj552510_78@163.com

**Keywords:** *Streptococcus suis* serotype 28, *Streptococcus suis* serotype 31, characterization, genome, antibiotic resistance

## Abstract

*Streptococcus suis* causes disease in pigs and is implicated increasingly in human disease worldwide. Although most clinical cases are associated with serotype 2, infections by other serotypes have sometimes been reported. Here, we sequenced the genome of a multidrug-resistant *S. suis* serotype 28 (strain 11313) and a multidrug-resistant *S. suis* serotype 31 (strain 11LB5). Strain 11313 was apathogenic in mouse infection models, whereas strain 11LB5 displayed ganglion demyelination, meningeal thickening, congestion, mononuclear cell infiltration, massive proliferation of cortical glial cells, and bacteria (>10^4^ CFU/g) in the spinal cord and ganglia in mice. Furthermore, immunohistochemistry found that the heavily infiltrated glial cells were astrocytes. Strain 11313 harbored the resistance genes *ant(6)-Ia*, *erm(B)*, *optrA*, *tet(l)*, *tet(o)*, and strain 11LB5 harbored the resistance genes *ant(6)-Ia*, *erm(B)*, *tet(40)*, *tet(o/w/32/o)*, *aac(6′)-aph(2″)*. Mouse studies showed that strain 11LB5 exhibited a similar virulence to serotype 2 strain 700794, highlighting the need for surveillance of the other serotype *S. suis* isolates, in addition to serotype 2, in farms. This is the first report of the aminoglycoside resistance gene *ant(6)-Ia* in *S. suis* from animals. This suggests that *S. suis* might serve as an antibiotic resistance reservoir, which spreads the resistance gene *ant(6)-Ia* or *optrA* to other streptococcal pathogens on farms.

## 1. Introduction

*Streptococcus suis*, an organism of the upper respiratory tract of pigs, can cause septicemia, meningitis, endocarditis, bronchopneumonia, arthritis and sudden death, and is divided into 29 serotypes (types 1–19, 21, 23–25, 27–31, and 1/2) based on capsular polysaccharide (CPS) according to recent studies to date [1,2,3,4]. *S. suis* infections are responsible for major economic losses in the swine industry and are important drivers of the spread of antibiotic resistance throughout the world [5]. Accurate *S. suis* serotyping is important for the epidemiologic control of pig infections, since the prevalent serotypes vary in different parts of the world [6]. 

In China, although *S. suis* serotype 2 is by far the most common isolated serotype and also the one most often related to disease, serotypes 1, 7, 9, and 14 can also cause disease [7]. Epidemiological surveys have shown the presence of many different serotypes including 3, 4, 5, 8, 11, 12, 13, and 28 [8,9,10] and different sequence types (STs) at Chinese pig farms [7]. However, although *S.suis* serotypes have been isolated from animals and humans worldwide [8,11,12,13], there are limited studies on the pathogenesis, genetic evolution, and drug resistance of *S. suis* and other serotypes. 

Here, we report the isolation and characterization of a β-hemolytic *S. suis* serotype 28 (strain 11313) and an α-hemolytic *S. suis* serotype 31 (strain 11LB5) from clinically ill pigs in Heilongjiang Province, China. We sequenced the complete genomes of both strains, comparing their *cps* gene cluster sequences with those of *S. suis* representative strains and evaluated the phylogenetic relationships among all *S. suis* representative serotype strains using serotype-specific gene polysaccharide polymerase (*wzy*). Pathogenicity and antibiotic resistance (AR) profiles of the strains were also determined. This study could help us better understand the diversity of the epidemic serotypes and the pathogenicity of serotypes other than serotype 2.

## 2. Materials and Methods

### 2.1. Animal Ethics Statements

This study was carried out in accordance with recommendations from the Guide for the Care and Use of Laboratory Animals of the Ministry of Science and Technology of China. The protocols were reviewed and approved by the Committee on the Ethics of Animal Experiments of the Harbin Veterinary Research Institute of the Chinese Academy of Agricultural Sciences. Mouse infection experiments (approval number SY-2013-MI-034) with *S. suis* were conducted within the animal biosafety level 2 facilities in the Harbin Veterinary Research Institute of the Chinese Academy of Agricultural Sciences (CAAS).

### 2.2. Bacterial Strains and Culture Conditions

Two novel *S. suis* strains were isolated from the brain or synovial fluid of two diseased pigs with neurological or arthritis symptoms from two different pig farms of Heilongjiang Province, China, in 2011. Briefly, brain or synovial fluid samples were cultured on sheep blood agar plates (OXOID) for 20 h at 37 °C. The isolated colonies were cultured for 18 h in Todd Hewitt broth (THB) (BD, Franklin, USA) at 37 °C, prior to extraction of genomic DNA by a genomic DNA extraction kit (Tigan, Beijing, China). The isolates were named strains 11313 and 11LB5, respectively.

Strains 11313 and 11LB5 were Gram-stained, and morphological characterization was performed on a general optical microscope (Primo Star, ZEISS, GmbH, Jena, Germany). The two strains were observed by transmission electron microscopy. Biochemical reactions were performed with BioMerieux api 20 STREP. 16S rRNA identification of each isolate was performed using the following primers: F 5′-AGA GTT TGA TCC TGG CTC AG-3′ and R 5′-TAC CTT GTT ACG ACT T-3′ [14]. PCR amplification was carried out using a program consisting of initial denaturation at 95 °C for 5 min, 25 cycles of denaturation at 94 °C for 1 min, annealing at 50 °C for 1 min, extension at 72 °C for 1 min, and a final extension at 72 °C for 10 min. Serological typing of strains 11313 and 11LB5 was performed by *Staphylococcal* Protein A (SPA) coagglutination test using 29 specific *S. suis* serotype antisera (Statens Serum Institute, Copenhagen, Denmark). Multilocus sequence typing (MLST) was applied to the indicated isolates involved seven housekeeping gene loci, including *cpn60, dpr, recA, aroA, thrA, gki,* and *mutS*. The allelic profile or sequence type (ST) of each isolate was identified in the *S. suis* MLST database (http://ssuis.mlst.net/, accessed on 18 March 2019).

### 2.3. Whole-Genome Sequencing Analysis

Genomic DNA of strains 11313 and 11LB5 were extracted from overnight cultures using a Bacterial DNA Extraction Kit (Tiangen, Beijing, China), according to the manufacturer’s instruction. The DNA concentration was determined by a Nanodrop 2000 Spectrophotometer (Thermo scientific, Waltham, MA, USA). For two isolates, the whole genomes were sequenced with a paired reads library with an average insert size (125 bp + 125 bp) on a HiSeq 2500 sequencer (Illumina, San Diego, CA, USA). The paired-end raw reads were corrected as clean reads utilizing a FASTX-Toolkit (http://hannonlab.cshl.edu/fastx_toolkit/, accessed on 19 September 2018), and then assembled using Velvet V1.2.03. Gene prediction was performed using Glimmer version 3.0. Predictions of tRNA and rRNA were conducted with tRNAS and RNAmmer, respectively. COG classification was conducted using CDD database and metabolic pathways were built through the KEGG database. Annotation of resistance genes was carried out using the online ResFinder database (https://cge.cbs.dtu.dk/services/ResFinder/, accessed on 17 May 2020). The *cps* locus sequence comparisons were performed by the software *MUSCLE* 3.8.31 [15] and BLASTN, respectively. Gene organization diagrams were drawn in the software Inkscape 0.48.1 (https://inkscape.org/en/, accessed on 24 February 2020). 

### 2.4. Phylogenetic Analysis

To construct a phylogenetic tree, we utilized serotype-specific gene *wzy* sequences from the two *S. suis* genomes isolated in the study. The nucleotide sequences of *wzy* gene of the indicated strains were aligned by *MUSCLE* 3.8.31 [15]. The unrooted neighbor-joining tree was generated from aligned *wzy* gene sequences by *MEGA7* [16], and evolutionary distances were estimated using the maximum composite likelihood method, with a bootstrap iteration of 1000. Data from the 29 *S. suis wzy* genes used for phylogenetic tree construction are listed in Table 1. 

### 2.5. Antibiotic Susceptibility Test

An antibiotic susceptibility test of strains 11313 and 11LB5 was performed by the drug sensitive paper diffusion method and BioMérieux VITEK 2, according to the standard methods in the Clinical and Laboratory Standards Institute (CLSI) guidelines (2016). The following antimicrobials were tested: ampicillin, ceftazidime, meropenem, cefoxitin, streptomycin, tetracycline, chloramphenicol, trimethoprim, nitrofurantoin, ciprofloxacin, spiramycin, Fosfomycin, and vancomycin. *Streptococcus pneumoniae* ATCC49619 was used as a control for all antimicrobial susceptibility testing.

### 2.6. Experimental Animal Infection

The virulence of *S. suis* strains 11313 and 11LB5 was evaluated in mouse studies. Briefly, 40 6-week-old female CD1 mice (11 mice/infected group, 7 mice/control group) were injected intraperitoneally (i.p.) with 0.25 mL containing *S. suis* strain 700794 (positive control), strain 11313 or 11LB5 (5 × 10^7^ CFU total), or sterile THB (negative control). Infected mice were monitored carefully for clinical symptoms and survival time for 10 days. Three mice from each group were euthanized humanely at 3 days post-inoculation (3 dpi), blood and tissue samples were collected. Then, 5 μL of blood or 10 mg tissue sample was plated and cultured on THB agar plates for 24 h. Tissue samples of mice were fixed in 10% formalin buffer and embedded in paraffin, cut in 2–3 µm thick slices and stained by hematoxylin and eosin (H&E), then observed by DM4000B upright fluorescence microscope (Leica, Frankfurt, Germany). To determine the type of glial cells that increased in the brain after infection, immunohistochemistry (IHC) was performed after deparaffinization and antigen retrieval (high pressure, 121 °C, 15 min), by addition of rabbit anti-mouse GFAP or Iba1 antibody to stain astrocytes or microgila (abcam, Cambridge, UK) and HRP-conjugated goat anti-rabbit antibody (Sigma, Saint Louis, MO, USA). Then, DAB (3, 3′-diaminobenzidine) was used to visualize the secondary antibody.

### 2.7. Statistical Analyses

Numerical data is expressed as the mean ± SD and were analyzed by GraphPad Prism software version 5.02 (GraphPad Software Inc., La Jolla, CA, USA). Differences between groups were assessed using *t*-tests or unpaired *t*-tests. A *p*-value less than 0.05 was considered statistically significant.

## 3. Results

### 3.1. Characteristics of S. suis Strains 11313 and 11LB5

**Figure 1:**
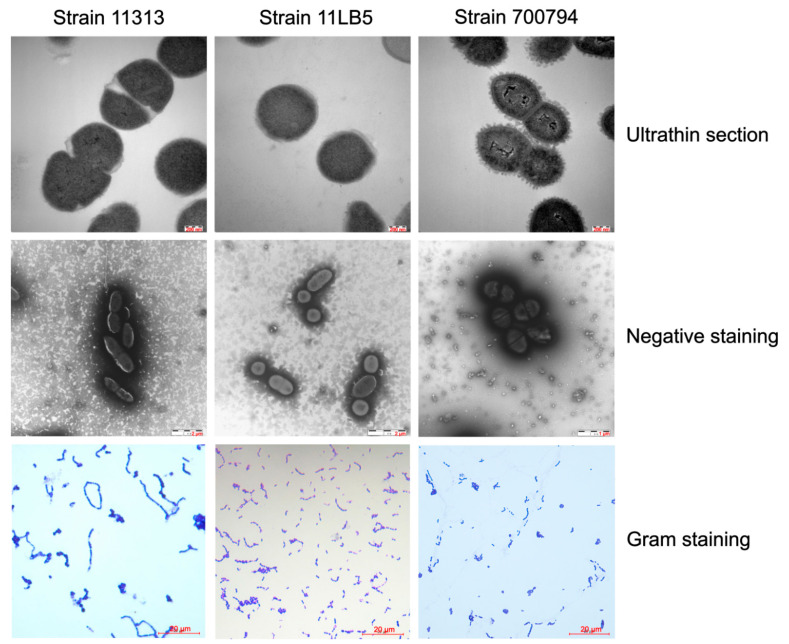
Transmission electron and Gram-staining micrographs of *S. suis* strains 11313 and 11LB5. *S. suis* 11313, 11LB5, and reference strains samples were collected for electron micrographs observation by HITACHI H-7650 transmission electron microscope. Ultrathin section, scale bar = 0.2 μm. Negative staining, 11313 and 11LB5, scale bar = 2 μm; 700794, scale bar = 1 μm. Gram staining, scale bar = 20 μm.

The two strains 11313 and 11LB5 were isolated from the brain or synovial fluid, respectively, of two diseased pigs from Heilongjiang Province, China, in 2011. Strains 11313 and 11LB5 formed slightly semitransparent or gray, smooth, wet, and glossy colonies after culturing for 24 h on sheep blood agar plates. The strains showed β-hemolysis and α-hemolysis, respectively, with 0.8 mm and 0.5 mm hemolytic rings. Strains 11313 and 11LB5 are Gram-positive cocci that occur in pairs, and short chains. Electron micrographs of ultrathin sections (Figure 1) show two strains were most likely encapsulated with 5 to 7 nm capsule thicknesses. Serotype 2 strain 700794 was used as a control and was encapsulated well with a thickness from 35 to 45 nm. The biochemical reactivity profiles of these two strains are in conformity to *S. suis* (data not shown). In addition, 16S rRNA and coagglutination identification indicated that the two isolates were *S. suis* of unknown serotype. The MLST analysis classified strains 11313 and 11LB5 as ST422 (allelic profile: 7, 58, 36, 54, 42, 37, 12) and ST421 (allelic profile: 1, 35, 2, 7, 1, 52, 28), respectively.

### 3.2. General Genomic Features of Strains 11313 and 11LB5 

To elucidate the genomic properties of the strains 11313 and 11LB5, we performed whole-genome sequencing for both strains. Strain 11313 sequencing yielded 4,460,968 reads and the genome consisted of a 2,261,105 bp chromosome with 41.15% G + C content, 1860 genes, 52 tRNAs, and 16 rRNAs (Table 2). Among the genes, 1802 matched homologous sequences in the nr database of NCBI, of which 1412 have clear functions. Meanwhile, strain 11LB5 sequencing yielded 6,553,398 reads and the assembled genome consisted of a 2,202,712 bp chromosome with 41.42% G + C content, 2078 genes, 49 tRNAs, and 8 rRNAs (Table 2). Among the 2078 genes, 1998 had homologous matches in the nr database, of which 1423 have clear functions. The genome sequences for strains 11313 and 11LB5 were deposited in GenBank under accession numbers NA647439 and NA647982, respectively. 

### 3.3. Structure of the Cps Gene Cluster in Strains 11313 and 11LB5

**Figure 2:**
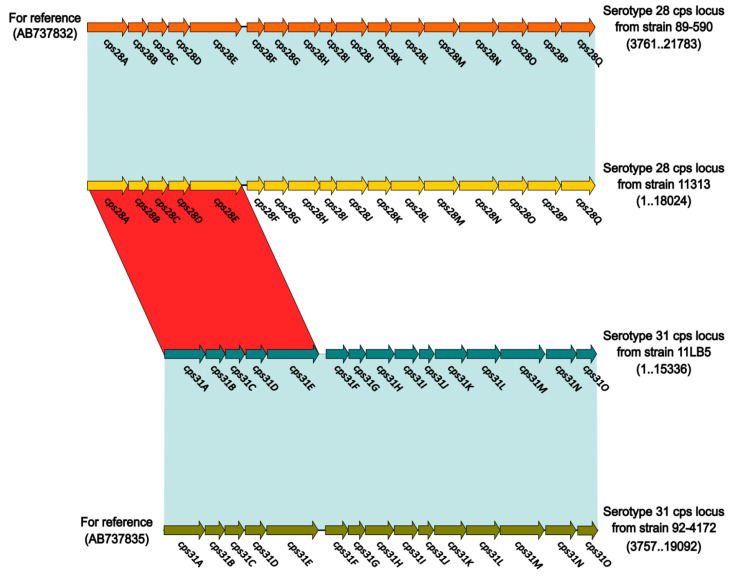
Linear comparison of complete sequences of *cps* gene locus sequences of *S. suis* strains 11313, 11LB5, and reference strains. Genes are denoted by arrows, and colored based on strains. Shaded regions denote homology of *cps* gene locus sequences (blue, ≥98% nucleotide identity; red, <86% nucleotide identity; .., to).

As shown in Figure 2, the *cps* gene cluster from strain 11313 is composed of 17 genes (*cpsA-cpsQ*) as described for the serotype 28 reference strain 89–590 (accession number AB737832). The *cps* locus from strain 11313 only had two nucleotide mutations relative to the *cps* locus from the reference strain 89–590, with 99.99% nucleotide identity and 100% coverage, and no gene deletions, insertions, or frameshifts were found. Similarly, the *cps* gene cluster of strain 11LB5 is composed of 15 genes (*cpsA-cpsO*) as described for the serotype 31 reference strain 92–4172 (accession number AB737835). The *cps* locus from strain 11LB5 had 98.76% nucleotide identity and 100% coverage from strain 92–4172, with no gene deletions, insertions or frameshifts. The two newly sequenced strains shared 84.6% nucleotide identity (*cpsA-cpsE*) and 30% *cps* locus coverage between them. Therefore, isolate 11313 was identified as *S. suis* serotype 28, and isolate 11LB5 as *S. suis* serotype 31.

### 3.4. Phylogenetic Analysis of Serotype-Specific Genes of Strains 11313 and 11LB5 

**Figure 3:**
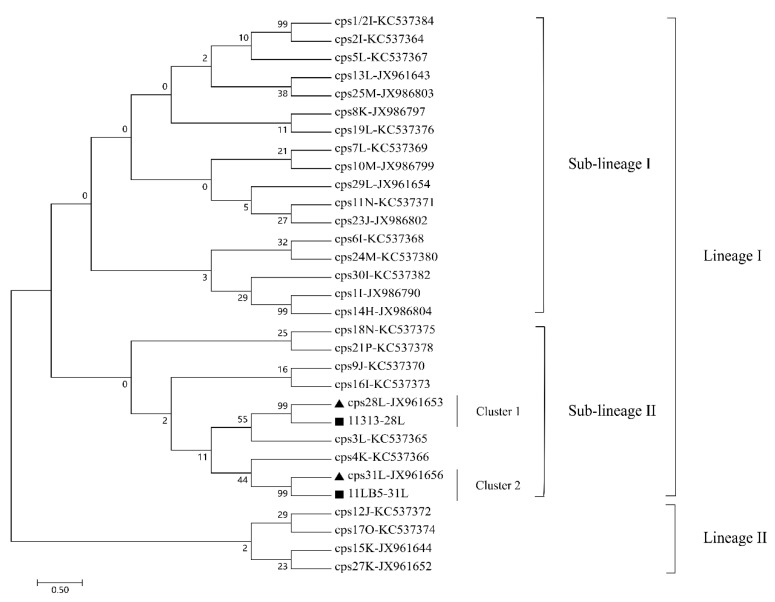
A neighbor-joining phylogenetic tree for the serotype-specific gene *wzy*. The degree of support (percentage) for all serotypes in the *wzy* gene, as determined by bootstrap analysis. The bar corresponds to the scale of sequence divergence; triangles indicate the reference sequences, while squares denote the genomes sequenced in this study.

To analyze the evolutionary relationship between strains 11313, 11LB5, and other *S. suis* strains, we constructed a phylogenetic tree based on the polysaccharide polymerase gene *wzy*. As shown in Figure 3, strains 11313 and 11LB5 are located on the lineage I of the tree and in the same sub-lineage, but they are in different clusters. The *wzy* gene from 11313 is included in cluster 1 (closest to *cps28L*), while 11LB5 is included in cluster 2 (closest to *cps31L*).

### 3.5. Antimicrobial Susceptibility Profiles

Multiple AR genes were found, including *ant(6)-Ia*, *erm(B), optrA, tet(l),* and *tet(o)* in strain 11313, and *aac(6′)-aph(2′’), ant(6)-Ia, erm(B), tet(40),* and *tet(o/w/32/o)* in strain 11LB5. Therefore, we decided to test both strains for susceptibility to ampicillin, ceftazidime, meropenem, cefoxitin, streptomycin, tetracycline, chloramphenicol, trimethoprim, nitrofurantoin, ciprofloxacin, spiramycin, Fosfomycin, and vancomycin (Table 3). Both strains were highly resistant to streptomycin, tetracycline, and spiramycin, with growth exclusion diameters ≤10 mm. Moreover, strain 11313 was moderately resistant to chloramphenicol (growth exclusion diameter of 16 mm) and resistant to trimethoprim. 

### 3.6. Virulence Evaluation of Strains 11313 and 11LB5 Isolates in Mice 

**Figure 4:**
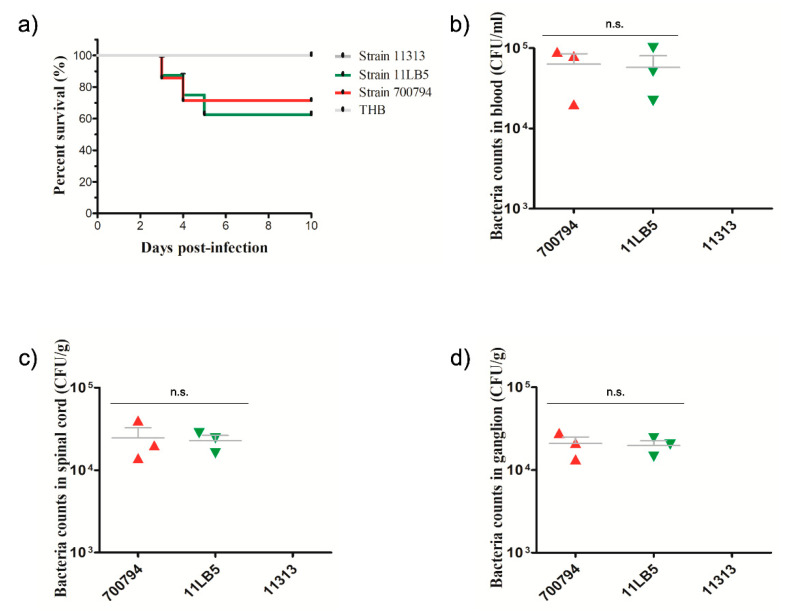
Virulence of *S. suis* strains 11303 and 11LB5 in a mouse model. Mice were challenged with the indicated strains, as described in Experimental Procedures. (**a**) Survival rates of mice inoculated with serotype 28 strain 11313, serotype 31 strain 11LB5, serotype 2 reference strain 700794, or Todd Hewitt broth (THB, control). Bacterial loads in (**b**) the blood (CFU/ml), (**c**) spinal cord (CFU/g of tissue), and (**d**) ganglia (CFU/g) of mice inoculated with strain 11LB5 or 700794 were not significantly different (*p* > 0.05). Results are expressed as means ± SD, and significance was determined using *t*-test and the unpaired *t*-test. n.s., not significantly different; green triangle: Strain 11LB5; red triangle: Strain 700794.

To evaluate the virulence of strains 11313 and 11LB5 in vivo, we injected six-week-old CD1 mice and tracked their survival and clinical symptoms. The mice infected with strain 11313 showed no clinical abnormalities and all survived, whereas most (10/11) of the mice infected with strain 11LB5 exhibited typical clinical symptoms including poor appetite, weight loss, rough coat, depression, shivering, inflammation of the cornea, and three of eight mice died, similar to the serotype 2 reference strain 700794 (Figure 4a). Bacteremia induced by strain 11LB5 was monitored by colony count after plating 5 μL blood on THA, revealing high levels (10^5^ CFU/mL) on 3 dpi (Figure 4b). Bacteria (>10^4^ CFU/g) from every mouse were also detected in the spinal cord and ganglia (Figure 4c,d). While no bacteria were detected in the blood, spinal cord, and ganglia of strain 11313. These results suggest that 11313 is an avirulent *S. suis* strain, whereas strain 11LB5 is virulent in mice. 

**Figure 5:**
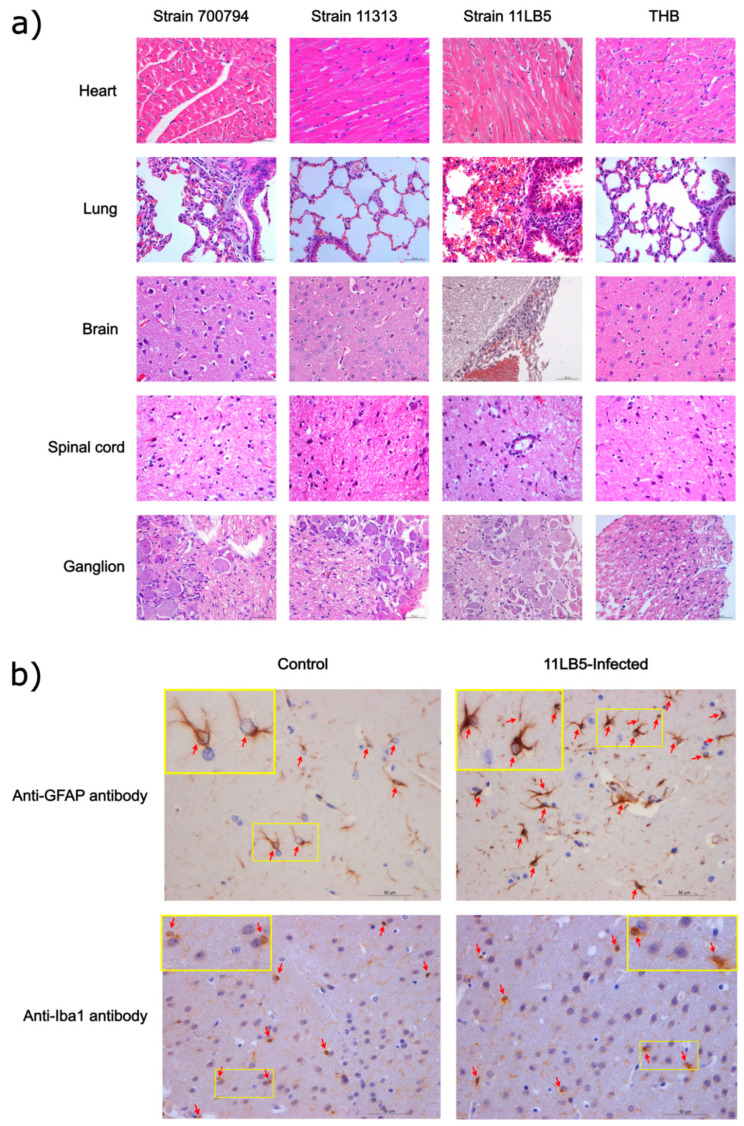
Histopathology and immunohistochemistry in organs of mice infected with *S. suis* strains 11303 or 11LB5. (**a**) Mice were infected with 5 × 10^7^ CFU of different *S. suis* strains or Todd Hewitt broth (THB, control), and heart, lung, brain, spinal cord, and ganglion samples were collected on 3 dpi for observation of histopathological lesions, scale bars = 50 μm; (**b**) Immunohistochemical analysis of brain samples from infected-11LB5 mice and control mice, using anti-GFAP antibody and anti-Iba1 antibody to stain the astrocyte (red arrows) and microglia (red arrows), quantified astrocytes and microgilas per field of vision in both infected and control samples (yellow rectangle), scale bars = 50 μm.

Histopathological lesions were determined in mice that were euthanized on 3 dpi (Figure 5a). In 700794-infected mice, there were no significant pathological changes in the heart and ganglia, but there was mild proliferation of glial cells in the spinal cord, the alveolar diaphragm was slightly widened, and exfoliated epithelial cells could be seen in the bronchus, with a significantly increased glial cells in the brain. However, in the 11LB5-infected mice, there was no significant change in the heart and the spinal cord. The main histopathological lesions observed in the lung were local congestion, a small amount of bleeding, a slight widening of the alveolar diaphragm, and a small number of epithelial cells of the bronchioles falling off. In the ganglia, some nerves were demyelinated and some cell nuclei disappeared. In the infected brains, there was local meningeal thickening, congestion with mononuclear cell infiltration, and increased glial cells could be seen in the cerebral cortex. In the 11313-infected mice, there were no significant pathological changes in any organs as compared with the controls. We performed IHC in order to identify which type of glial cells were increased massively in the cerebral cortex of 11LB5-infected brains. As showed in Figure 5b, there were more than four astrocytes per field of vision in the infected group, but two astrocytes per field of vision in control group. However, there were two microgilas per field of vision both in the infected group and in the control group. Therefore, astrocytes were the type of glial cells that increased massively in the 11LB5-infected brains.

## 4. Discussion

Serotyping of *S. suis* is one of the most useful methods to understand the epidemiology of a particular outbreak and monitor the prevalence of potentially hazardous strains [17]. CP synthesis genes are clustered on a single locus of the chromosome in *S. suis* [18], and their general organization suggests that they may be synthesized by a Wzy-dependent pathway [19]. In this study, we sequenced and analyzed the *cps* gene clusters of two encapsulated strains isolated from clinically ill pigs, but their capsule thickness was thin as compared with the encapsulated strain 700794 and strains from a previous study [13], which may explain why the serum coagglutination tests were unable to determine their serotypes. The nucleotide sequence of the *cpsA-cpsD* regions are conserved in all serotypes and located on the 5′-side of the *cps* gene clusters [20]. In our study, the *cpsA-cpsE* region of both strains share approximately 85% sequence identity, but other regions of the *cps* gene clusters had low similarity. Our isolates are serotype 28 and serotype 31, respectively, which were reported rarely isolated from the farms. In the two sick pigs, the strain 11LB5 was the only *S. suis* isolated from the first sick pig, indicating that it was responsible for disease in this pig. In contrast, a second *S. suis* strain of virulent serotype 7 was isolated along with strain 11313 in the second pig (data not shown). Presumably, it was the virulent serotype 7 strain that caused disease in the second pig, although we cannot at this stage rule out an effect of strain 11313. The pathogenicity of strain 11313 to pigs will be further investigated in future studies.

MLST is a highly discriminatory method used to characterize bacterial population structure, which has been performed to investigate genotypes and microevolution of *S. suis* since 2002 [21]. Most pathogenic *S. suis* serotype 2 strains belong to ST1, as shown previously [7]. The virulent strain 11LB5 belongs to the relatively uncommon ST421, which has been isolated repeatedly at the same farm in recent years (data not shown). As ST421 has been circulating on this farm for years, it highlights the need for surveillance of *S. suis* isolates other than ST1. We have shown that strain 11LB5 could cause sepsis, similar to the *S. suis* serotype 2 used in this study and *S. suis* serotype 31 from human infections in Thailand [13]. In this study, a virulent serotype 31 strain 11LB5 was isolated from the farm, suggesting that farms may act as a pathogen reservoir, contributing to the spread of serotype 31 strain to humans. Therefore, increased awareness about *S. suis* infection in swine is needed for the benefit of public health.

This is believed to be the first report of *S. suis* infection in the spinal cord and ganglia of mice, which may be related to *S. suis* that can cause neurological symptoms in the animals. Glial cells may play a crucial role in host–pathogen interactions during *S. suis* infection of the central nervous system and are considered to possess important functions during inflammation and brain injury in bacterial meningitis [22]. A massive increase in glial cells was often found in the cerebral cortex of infected mice with meningitis caused by *S. suis* in our studies (Figure 5a). Furthermore, we identified these glial cells to be astrocytes by using IHC. Factors produced by activated astrocytes can target neighboring cells and promote leukocyte recruitment, resulting in local amplification of the inflammatory responses [23]. Thus, our study suggests that astrocytes might be more important in the process of inflammation and removal of foreign pathogens from the brain.

Antimicrobials have been widely used in animals for the prevention and treatment of bacterial disease as well as growth promoters. Aminoglycosides, the third most common class of antimicrobials used worldwide (after sulfonamides and beta-lactams) are a recommended alternative for the treatment of infections in humans [24]. *Ant(6)-Ia* genes encoding aminoglycoside O-nucleotidyltransferases have been reported in resistant strains of Gram-negative bacteria including *Campylobacter jejuni*, *Campylobacter coli, Enterococci,* and *Lactobacillus* from humans or animals [25,26,27,28]. However, in Gram-positive bacteria, the *ant(6)-Ia* gene has only been found in isolates from humans [29]. Our study is the first report of *ant(6)-Ia* genes in the zoonotic pathogen *S. suis* isolated from animals, suggesting that *S. suis* may act as an antibiotic resistance reservoir, contributing to the spread of the resistance gene *ant(6)-Ia* to other streptococcal pathogens on farms. The importance of this possible intersection between animal and human resistome highlights the importance of surveillance of AR genes in general. Both *S. suis* strains were highly resistant to tetracycline, with growth exclusion diameters of ~6 mm, which were observed in strains carrying *tet(l), tet(o), tet(40),* and *tet(o/w/32/o)* genes as reported in previous studies [5,30]. AR gene *optrA*, which represents an important acquired resistance mechanism to last resort antibiotic oxazolidinones and have been proven to be functional in oxazolidinone resistance in a previous study [31]. In addition, *optrA* not only mediates resistance to oxazolidinones (including linezolid and tedizolid), but also mediates resistance to phenylpropanols (including florfenicol and chloramphenicol) and can spread horizontally [32]. The *optrA* gene of 11313 showed 99.9% identity to *S. suis* HA1003 (CP030125), and 99.84% identity to *E. faecalis* SJ117 (MN257069) and *C. jejuni* ZS007 (CP048771). 11313 was moderately resistant to chloramphenicol, which was observed in strains carrying *optrA* gene as reported in previous studies [33,34]. The *erm(B)* gene is present in a variety of Gram-positive bacteria, including *enterococci*, *streptococci*, and *staphylococci* [35]. AR gene *erm (B)* served as the most frequent genotype that contributed to macrolide resistances [36]. In our study, strains 11313 and 11LB5 carry *erm (B)* gene and appear to be highly resistant to macrolide antibiotics spiramycin. 

In conclusion, we isolated, identified, and sequenced a multidrug-resistant *S. suis* serotype 28 and a multidrug-resistant *S. suis* serotype 31. Both isolates harbored the aminoglycoside resistance gene *ant(6)-Ia*, which is the first report of the *ant(6)-Ia* gene in zoonotic pathogen *S. suis* isolated from animals. Moreover, it is also the first report of *S. suis* infection in the spinal cord and ganglion of mice, and we found that the heavily infiltrated glial cells in the cerebral cortex were astrocytes. In summary, our results highlight the need for surveillance of other multidrug-resistant *S. suis* serotypes beyond the common serotype 2 on Chinese farms.

## 5. Conclusions

A multidrug-resistant *S. suis* serotype 28 and a multidrug-resistant *S. suis* serotype 31 were characterized, which will contribute to a better understanding of the genetic diversity of *S. suis* beyond serotype 2.

## Figures and Tables

**Table 1:** *Streptococcus suis* serotype-specifc *wzy* genes used in this study.

Strain	Serotype	*wzy* Genes	Accession No.
11313	28	*cps28L*	NA647439
11LB5	31	*cps31L*	NA647982
2651	1/2	*cps1/2I*	KC537384
5428	1	*cps1I*	JX986790
R735	2	*cps2I*	KC537364
4961	3	*cps3L*	KC537365
6407	4	*cps4K*	KC537366
11538	5	*cps5L*	KC537367
2524	6	*cps6I*	KC537368
8074	7	*cps7L*	KC537369
14636	8	*cps8K*	JX986797
22083	9	*cps9J*	KC537370
4417	10	*cps10M*	JX986799
12814	11	*cps11N*	KC537371
8830	12	*cps12J*	KC537372
10581	13	*cps13L*	JX961643
13730	14	*cps14H*	JX986804
NCTC 10446	15	*cps15K*	JX961644
2726	16	*cps16I*	KC537373
93A	17	*cps17O*	KC537374
NT77	18	*cps18N*	KC537375
42A	19	*cps19L*	KC537376
14A	21	*cps21P*	KC537378
89-2479	23	*cps23J*	JX986802
88-5299A	24	*cps24M*	KC537380
89-3576-3	25	*cps25M*	JX986803
89-5259	27	*cps27K*	JX961652
89-590	28	*cps28L*	JX961653
92-1191	29	*cps29L*	JX961654
92-1400	30	*cps30I*	KC537382
92-4172	31	*cps31L*	JX961656

**Table 2:** Characteristics of *Streptococcus suis* genomes analyzed in this study.

Strain	Serotype	MLST Type	Accession No.	Size (bp)	GC (%)	Genes	Proteins	Antibiotic Resistance Genes	Virulence Phenotype
11313	28	ST422	NA647439	2,261,105	41.45	1860	1802	*erm(B),optrA, tet(l), tet(o), ant(6)-Ia*	*gdh+/mrp-/sly-/epf-*
11LB5	31	ST421	NA647982	2,202,712	41.42	2078	1998	*aac(6′)-aph(2′’), ant(6)-Ia, erm(B), tet(40), tet(o/w/32/o)*	*gdh+/mrp-/sly-/epf-*

**Table 3:** Antimicrobial drug susceptibility profiles.

Category	Antibiotic	K-B(mm)/Antimicrobial Susceptibility *			MIC (mg/L) /Antimicrobial Susceptibility *		
		Strain 11313	Strain 11LB5	Strain 700794	Strain 11313	Strain 11LB5	Strain 700794
Penicillin	Ampicillin	15/S	24/S	20/S	<8/S	<4/S	<4/S
Cephalosporin	Ceftazidime	26/S	21/S	26/S	<4/S	<4/S	<4/S
Carbapenem	Meropenem	38/S	50/S	40/S	<1/S	<1/S	<1/S
Cephamycin	Cefoxitin	25/S	34/S	30/S	<8/S	<4/S	<4/S
Aminoglycoside	Streptomycin	10/R	6/R	10/R	>512/R	>512/R	>512/R
Tetracycline	Tetracycline	6/R	6/R	20/S	>512/R	>512/R	<8/S
Phenicol	Chloramphenicol	16/I	40/S	21/S	16/I	<4/S	<8/S
Folate pathway	Trimethoprim	10/R	28/S	20/S	>32/R	<0.5/S	<1/S
Nitrofuran	Nitrofurantoin	28/S	32/S	30/S	<16/S	8/S	8/S
Fluoroquinolone	Ciprofloxacin	6/R	22/S	30/S	>216/R	<1/S	<0.5/S
Macrolide	Spiramycin	6/R	6/R	23/S	>512/R	>512/R	8/S
Fosfomycin	Fosfomycin	25/S	32/S	26/S	<64/S	<32/S	<64/S
Peptide antibiotics	Vancomycin	15/S	25/S	20/S	8/S	4/S	4/S
oxazolidinones	linezolid	32/S	34/S	44/S	<0.5/S	<0.5/S	0.25/S

* S, sensitive; I, intermediate; R, resistant.

## Data Availability

Data are contained within the article.
